# Data on the selection of biostimulating agents for the bioremediation of soil simultaneously contaminated with lindane and zinc

**DOI:** 10.1016/j.dib.2018.08.203

**Published:** 2018-09-06

**Authors:** Mikel Anza, Oihane Salazar, Lur Epelde, Carlos Garbisu

**Affiliations:** NEIKER-Tecnalia, Department of Conservation of Natural Resources, Soil Microbial Ecology Group, c/ Berreaga 1, E-48160 Derio, Spain

## Abstract

The bioremediation of contaminated soil often involves the addition of organic/inorganic amendments and mobilizing agents (e.g. surfactants, detergents), in order to stimulate the growth and degrading activity of soil microbial populations and increase contaminant bioavailability. For this data article we carried out an experiment to select biostimulating agents for the bioremediation of soil simultaneously contaminated with lindane (HCH, 10 mg kg^−^^1^ DW soil) and Zinc (Zn, 1500 mg kg^−1^ DW soil). To this purpose, a factorial design was used to test the effect of three organic amendments (i.e. hen manure, composted horse manure, cow slurry) and three mobilizing agents (i.e. sodium dodecylbenzenesulfonate (SDS), rhamnolipids and Tween-80) on the reduction of total HCH and bioavailable Zn concentration in soil. Similarly, the effect of the addition of cyclohexane, as chemical inducer of HCH degradation, was also studied. The addition of SDS, rhamnolipids and Tween-80 significantly reduced HCH concentration in soil, regardless of the presence of other biostimulating agents. When added individually, the three organic amendments (hen manure, composted horse manure, cow slurry) significantly reduced bioavailable Zn concentration in soil. These data provide useful information for the bioremediation, through biostimulation, of soils simultaneously contaminated with HCH and Zn.

**Specifications table**

**Table:** 

Subject area	*Environmental sciences.*
More specific subject area	*Soil pollution, bioremediation, biostimulation.*
Type of data	*Table and Figures.*
How data was acquired	*A fractional factorial design was used to select biostimulating agents for the bioremediation of soil simultaneously contaminated with lindane and zinc.*
Data format	*Analyzed. Soil contaminant concentrations.*
Experimental factors	*Seven biostimulating agents were added to soil artificially contaminated with lindane and zinc: hen manure, composted horse manure, cow slurry, sodium dodecylbenzenesulfonate-SDS, rhamnolipids, Tween-80 and cyclohexane.*
Experimental features	*Lindane concentration in soil was determined by gas chromatography. Bioavailable zinc concentration in soil was measured by atomic absorption spectroscopy.*
Data source location	*Derio, Spain.*
Data accessibility	*Data are available in the article.*
Related research article	*No.*

**Value of the data**

• Data are useful for the bioremediation through biostimulation of soils simultaneously contaminated with lindane and zinc.
• Data are useful for the selection of organic amendments (hen manure, composted horse manure, cow slurry) and mobilizing agents in soil bioremediation.

## 1 Data

**Fig. 1:**
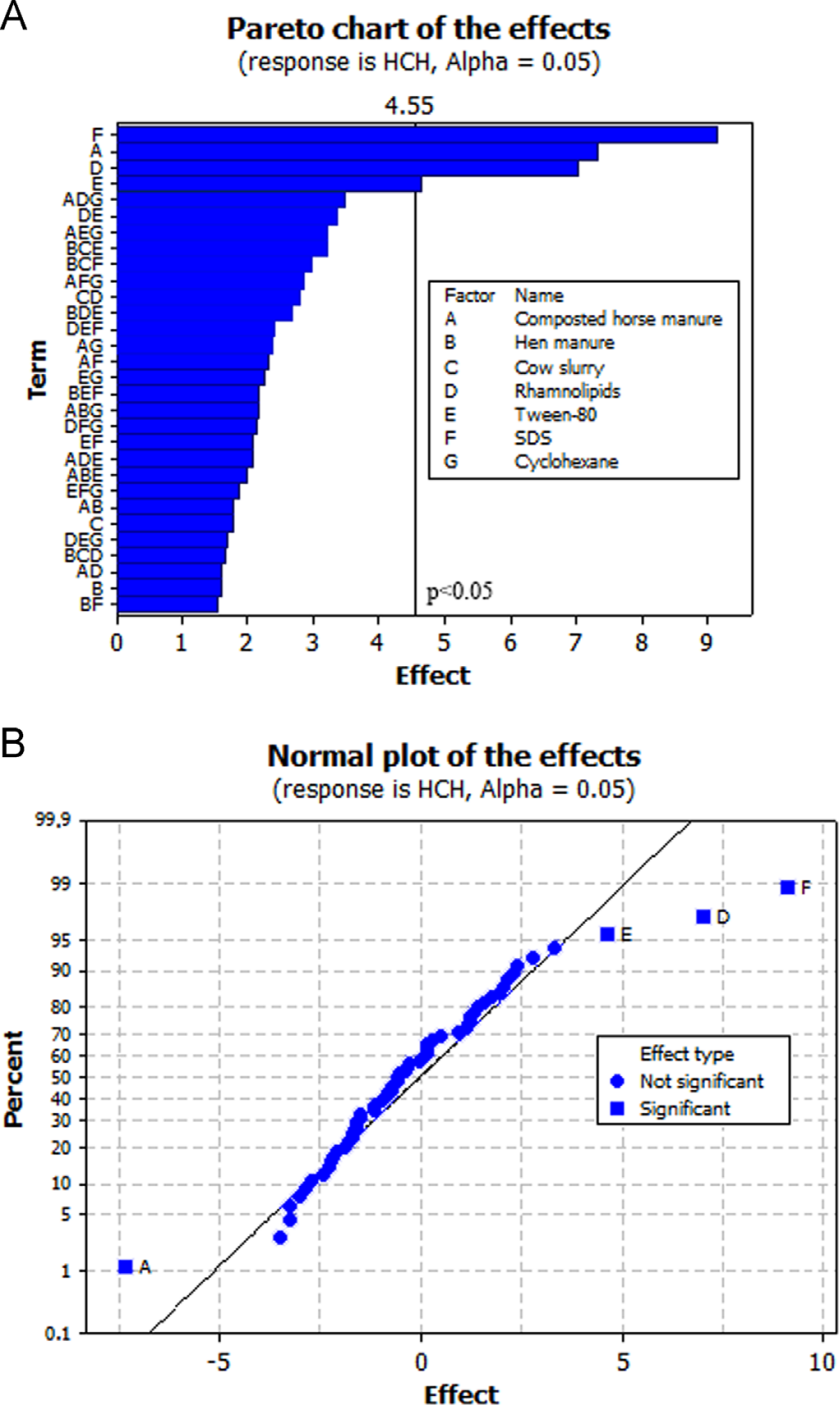
Pareto chart (A) and normal plot (B) for total HCH concentrations in soil.

**Fig. 2:**
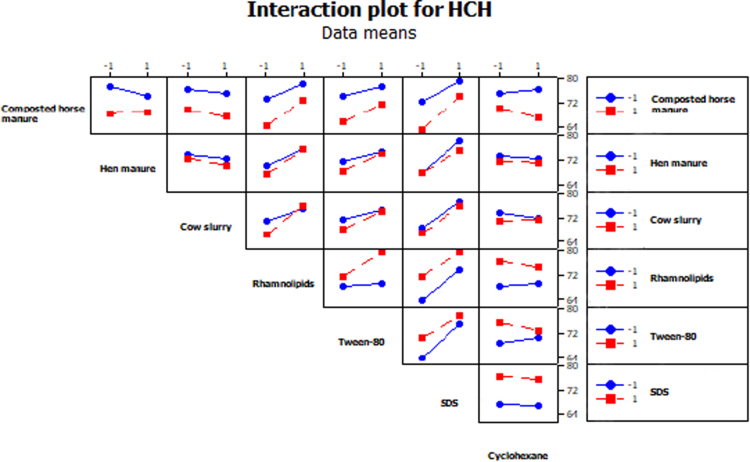
Interaction plot among seven factors for total HCH concentrations in soil.

**Fig. 3:**
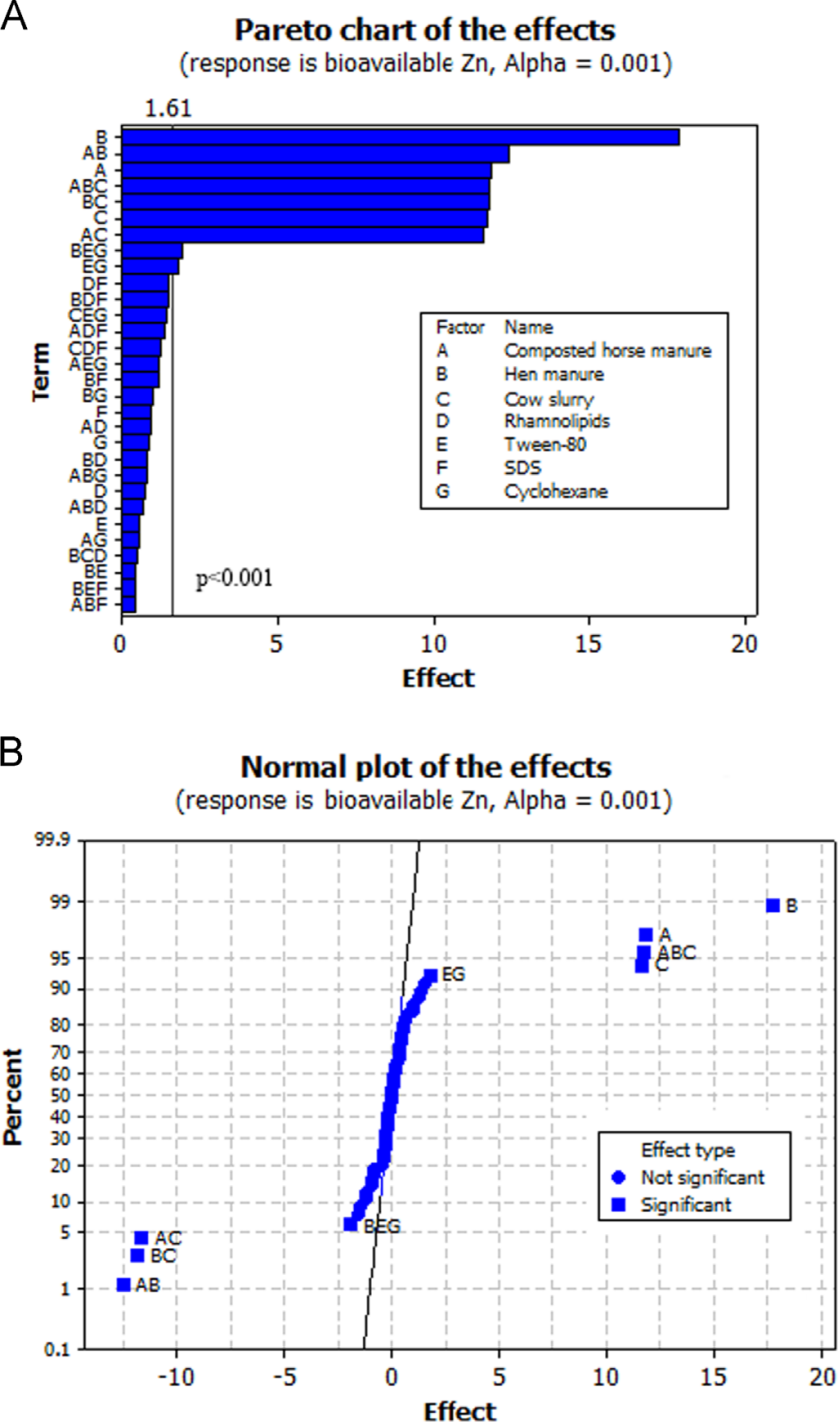
Pareto chart (A) and normal plot (B) for bioavailable Zn concentrations in soil.

**Fig. 4:**
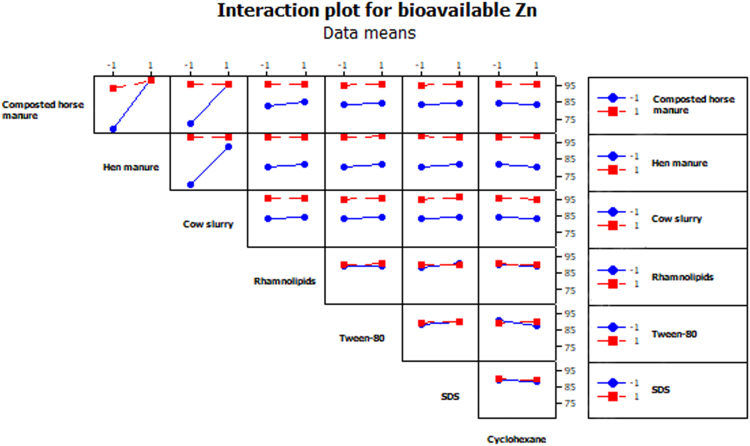
Interaction plot among seven factors for bioavailable Zn concentrations in soil.

The objective of the present work was to select biostimulating agents (i.e. *organic amendments* to promote the growth and degrading activity of soil microbial populations; *mobilizing agents* to increase HCH bioavailability; and *a chemical inducer* to stimulate HCH degradation) for the bioremediation of soil simultaneously contaminated with lindane (10 mg kg^−1^ DW soil) and Zn (1500 mg kg^−1^ DW soil). To this purpose, a fractional factorial experiment with seven factors [2^(7−1)^, Table 1] was used. Table 1 summarizes the 64 runs of the factorial design used here. Our experiment revealed that three factors (in order of importance: SDS, rhamnolipids and Tween-80) significantly reduced total HCH concentration in soil after 10 weeks of incubation (Fig. 1a: pareto chart; Fig. 1b: normal plot of the effects, α = 0.05; Table 1). In fact, the analysis of interactions between factors showed that the addition of SDS, rhamnolipids and, to a lesser extent, Tween-80 reduced total HCH concentration in soil, independently of the presence of the other factors (Fig. 2). Regarding bioavailable Zn concentration in soil, the three organic amendments used here (in order of importance: hen manure, composted horse manure and cow slurry) led to lower values of this parameter (Fig. 3, α = 0.001; Table 1). The analysis of the interactions between factors (Fig. 4) showed that the addition of organic amendments was effective only when they were added individually and not in combination.

**Table 1:** Fractional factorial design defined with seven factors (i.e. seven biostimulating agents) and 64 runs. [HCH] and [Zn]: % reduction in total HCH and bioavailable Zn concentration after 10 weeks of incubation.

Run no.	Horse compost	Hen manure	Cow slurry	Rhamnolipids	Tween-80	SDS	Cyclohexane	[HCH] %	[Zn] %
1	−1	−1	−1	−1	−1	−1	+1	71.8	33.7
2	+1	−1	−1	−1	−1	−1	−1	65.9	91.9
3	−1	+1	−1	−1	−1	−1	−1	67.3	98.7
4	+1	+1	−1	−1	−1	−1	+1	54.1	98.4
5	−1	−1	+1	−1	−1	−1	−1	65.9	90.4
6	+1	−1	+1	−1	−1	−1	+1	54.2	89.6
7	−1	+1	+1	−1	−1	−1	+1	58.1	99.0
8	+1	+1	+1	−1	−1	−1	−1	46.4	98.3
9	−1	−1	−1	+1	−1	−1	−1	71.7	51.2
10	+1	−1	−1	+1	−1	−1	+1	64.7	91.5
11	−1	+1	−1	+1	−1	−1	+1	70.8	99.4
12	+1	+1	−1	+1	−1	−1	−1	59.3	98.0
13	−1	−1	+1	+1	−1	−1	+1	70.5	89.0
14	+1	−1	+1	+1	−1	−1	−1	61.6	91.6
15	−1	+1	+1	+1	−1	−1	−1	73.7	98.4
16	+1	+1	+1	+1	−1	−1	+1	68.7	97.9
17	−1	−1	−1	−1	+1	−1	−1	76.4	36.9
18	+1	−1	−1	−1	+1	−1	+1	57.2	93.7
19	−1	+1	−1	−1	+1	−1	+1	72.7	98.9
20	+1	+1	−1	−1	+1	−1	−1	62.6	98.6
21	−1	−1	+1	−1	+1	−1	+1	71.8	88.7
22	+1	−1	+1	−1	+1	−1	−1	54.7	92.6
23	−1	+1	+1	−1	+1	−1	−1	70.9	98.3
24	+1	+1	+1	−1	+1	−1	+1	63.6	97.9
25	−1	−1	−1	+1	+1	−1	+1	78.4	51.7
26	+1	−1	−1	+1	+1	−1	−1	70.7	93.3
27	−1	+1	−1	+1	+1	−1	−1	78.2	98.9
28	+1	+1	−1	+1	+1	−1	+1	72.3	97.8
29	−1	−1	+1	+1	+1	−1	−1	76.5	93.8
30	+1	−1	+1	+1	+1	−1	+1	66.6	92.1
31	−1	+1	+1	+1	+1	−1	+1	81.0	98.9
32	+1	+1	+1	+1	+1	−1	−1	78.5	98.4
33	−1	−1	−1	−1	−1	+1	−1	82.6	52.8
34	+1	−1	−1	−1	−1	+1	+1	79.0	91.6
35	−1	+1	−1	−1	−1	+1	+1	77.5	98.2
36	+1	+1	−1	−1	−1	+1	−1	67.2	98.3
37	−1	−1	+1	−1	−1	+1	+1	80.3	91.0
38	+1	−1	+1	−1	−1	+1	−1	70.1	94.9
39	−1	+1	+1	−1	−1	+1	−1	71.1	97.6
40	+1	+1	+1	−1	−1	+1	+1	73.6	98.3
41	−1	−1	−1	+1	−1	+1	+1	82.2	39.7
42	+1	−1	−1	+1	−1	+1	−1	73.8	93.3
43	−1	+1	−1	+1	−1	+1	−1	78.7	98.2
44	+1	+1	−1	+1	−1	+1	+1	72.6	97.7
45	−1	−1	+1	+1	−1	+1	−1	76.8	96.4
46	+1	−1	+1	+1	−1	+1	+1	66.2	92.0
47	−1	+1	+1	+1	−1	+1	+1	83.2	98.4
48	+1	+1	+1	+1	−1	+1	−1	69.1	98.3
49	−1	−1	−1	−1	+1	+1	+1	79.0	48.1
50	+1	−1	−1	−1	+1	+1	−1	70.0	92.1
51	−1	+1	−1	−1	+1	+1	−1	76.2	99.0
52	+1	+1	−1	−1	+1	+1	+1	72.6	98.1
53	−1	−1	+1	−1	+1	+1	−1	73.6	93.0
54	+1	−1	+1	−1	+1	+1	+1	65.7	95.3
55	−1	+1	+1	−1	+1	+1	+1	71.7	98.9
56	+1	+1	+1	−1	+1	+1	−1	67.0	98.1
57	−1	−1	−1	+1	+1	+1	−1	79.7	45.4
58	+1	−1	−1	+1	+1	+1	+1	70.8	93.8
59	−1	+1	−1	+1	+1	+1	+1	75.4	99.0
60	+1	+1	−1	+1	+1	+1	−1	100.0	97.4
61	−1	−1	+1	+1	+1	+1	+1	100.0	92.6
62	+1	−1	+1	+1	+1	+1	−1	100.0	94.0
63	−1	+1	+1	+1	+1	+1	−1	76.6	99.0
64	+1	+1	+1	+1	+1	+1	+1	66.8	97.9

## 2 Experimental design, materials, and methods

Soil was collected (upper 20 cm) from a local meadow and, then, air-dried for one week. The soil was a clay–loam, with a pH of 6.2 and an organic matter content of 6.3%. After sieving (<5 mm), the soil was artificially contaminated with 10 mg γ-hexachlorocyclohexane kg^−1^ DW (dry weight) soil (Aldrich 233390, lindane, HCH) and 1500 mg Zn kg^−1^ DW soil (Aldrich 14436, zinc nitrate hexahydrate) as follows: a stock solution of HCH was prepared by dissolving 100 mg HCH in 250 mL acetone. This solution was then diluted eight fold. Finally, 200 mL of this solution per kg DW soil were sprayed onto the study soil and, subsequently, thoroughly mixed for homogenization. Acetone was left to evaporate for 2 h. After that, zinc nitrate hexahydrate (68.22 g) was diluted in 1 L deionized water. Similarly, 100 mL of this solution per kg DW soil were sprayed onto the study soil and, subsequently, thoroughly mixed for homogenization. Artificially contaminated soil was stabilized for two months at room temperature.

A fractional factorial design [2^(7−1)^] was used in this experiment, in order to reduce the number of required experimental microcosms (see below) and to be able to detect potential interactions between the seven independent factors (i.e. biostimulating agents) studied here. A total of 64 microcosms, each of them consisting of 100 g DW soil placed in a 250 mL conical flask, were established and allowed to precondition, under the experimental conditions, for 1 week following Epelde et al. [1]. Experimental soil was maintained at 60% of its water holding capacity (WHC) throughout the experiment. The seven biostimulating agents used here were: hen manure (15% DW soil), composted horse manure (15% DW soil), cow slurry (15% DW soil), sodium dodecylbenzenesulfonate (50 mg kg^−1^ DW soil, Sigma-Aldrich 289957), rhamnolipids [2] (10 mg kg^−1^ DW soil, Sigma-Aldrich R90-10G), Tween-80 [2], [3] (50 mg kg^−1^ DW soil, Sigma-Aldrich P1754), and cyclohexane (100 µL, Sigma-Aldrich, 227048). After 10 weeks of incubation at room temperature and in darkness, total HCH and bioavailable Zn (i.e. 0.01 M CaCl_2_-extractable) concentrations in soil were determined. We quantified bioavailable Zn concentration as it corresponds to that fraction of the total metal concentration that can interact with the biological target [4] and, in consequence, could negatively affect HCH-degrading microbial populations. Lindane was extracted from soil by sonicating 1.5 g of soil in 5 mL hexane/acetone (50:50 v/v), and then quantified by gas chromatography (Agilent 6890 N): µECD detector, HP-ULTRA 2 (25 m × 0.200 mm × 0.33 µm) column; injection volume = 2 µl in split mode; injector temperature = 250 °C; temperature ramp: 150 °C (2 min) – 20 °C min^−1^ – 300 °C (3 min); and flow of carrier gas (helium) = 3 mL min^−1^. Extractable-Zn (0.01 M CaCl_2_-extractable Zn), as an estimation of bioavailable Zn, was determined by atomic absorption spectroscopy (VARIAN), following Houba et al. [5]. The normal distribution of the data was confirmed following the Kolmogorov–Smirnov test.
